# Solid-state fermentation of *Apocynum venetum* L. by *Aspergillus niger*: Effect on phenolic compounds, antioxidant activities and metabolic syndrome-associated enzymes

**DOI:** 10.3389/fnut.2023.1125746

**Published:** 2023-02-27

**Authors:** Cha Cao, Dengfan Lin, Yingjun Zhou, Na Li, Yiwen Wang, Wenbeng Gong, Zuohua Zhu, Chengwei Liu, Li Yan, Zhenxiu Hu, Yuande Peng, Chunliang Xie

**Affiliations:** ^1^Institute of Bast Fiber Crops, Chinese Academy of Agricultural Sciences, Changsha, China; ^2^College of Life Sciences, Hunan Normal University, Changsha, China; ^3^Hunan Engineering Laboratory for Good Agricultural Practice and Comprehensive Utilization of Famous-Region Medicinal Plants, College of Bioscience and Biotechnology of Hunan Agricultural University, Changsha, China; ^4^Key Laboratory for Enzyme and Enzyme-Like Material Engineering of Heilongjiang, College of Life Science, Northeast Forestry University, Harbin, Heilongjiang, China

**Keywords:** biotransformation, *Aspergillus niger*, *Apocynum venetum* L., antioxidant activities, metabolic syndrome-associated enzymes, phenolic compounds

## Abstract

This study aimed to evaluate the effect of solid-state fermentation (SSF) with *Aspergillus niger* on the total phenolic content (TPC), the total flavonoid content (TFC), individual phenolic contents, and antioxidant and inhibitory activities against metabolic syndrome-associated enzymes in an ethanol extract from *Apocynum venetum* L. (AVL). TPC, TFC, and the contents of quercetin and kaempferol during SSF were 1.52-, 1.33-, 3.64-, and 2.22-fold higher than those of native AVL in the ethyl acetate (EA) subfraction of the ethanol extract. The ABTS·^+^, DPPH· scavenging, and inhibitory activities against α-glucosidase and pancreatic lipase were found to be highest in the EA subfraction. Fermentation significantly increased the ABTS radical cation, DPPH radical scavenging, and pancreatic lipase inhibitory activities by 1.33, 1.39, and 1.28 times, respectively. TPC showed a significantly positive correlation with antioxidant activities or inhibition against metabolic syndrome-associated enzymes. This study provides a theoretical basis for producing tea products with enhanced antioxidant, antidiabetic, and antihyperlipidemic activities.

## 1. Introduction

*Apocynum venetum* L. (AVL) has been used in traditional Chinese medicine and is widely grown in a saline–alkaline desert and on river banks (1). Due to the flavonoid-rich content in its leaves, it has the potential capacity to cure angiocardiopathies by lowering blood pressure, preventing hyperlipidemia, treating depression, and calming nerves (2–4). AVL-based medicines and tea beverages have attracted great interest in China, Japan, and the USA (2).

In previous studies, the main active fractions of AVL are phenolic acid, flavonoid (hyperoside and isoquercitrin), and flavan-3-ol components (5, 6). Phenolics exist in both soluble and insoluble-bound forms (7). Several previously published literature studies reported that the content of insoluble phenolic compounds in some agricultural by-products is listed in increasing order: 16% in pomegranate peel, 25% in pomegranate seed, 47% in chestnut shell, 53% in black carrot pomace, 57% in blueberry seed meal, 61% in blackberry seed meal, 63% in black raspberry seed meal, 63% in watermelon peel, 67% in melon peel, 68% in mango seed, 70% in pear peel, 79% in kiwi peel, 80% in orange peel, 82% in banana peel, and 88% in apple peel (8–11). Soluble phenolic compounds (PC) in plants can be extracted using organic solvents, such as ethanol, acetone, and ethyl acetate (EA) (12). However, insoluble phenolic components are bound to the cell structure *via* ester or glycosidic linkages and cannot be effectively extracted (11). This leads to low or limited utilization efficiency of these bioactive substances in materials (13). Thus, fermentation technologies can effectively improve their bioavailability. Nowadays, microbial fermentation with α-amylase, cellulase, β-glucosidase, and xylanase secreted by microorganisms can release phenolics (14). *Monascus anka, Bacillus* sp., and *Aspergillus oryzae* have been widely applied in oats (*M. anka*), black rice bran (*A. oryzae*), and guava leaves (*M. anka* and *Bacillus* sp.) on the release of PC and enhancement of bioactivities (15–17). *Aspergillus niger* (*A. niger*) enhanced the antioxidant activity of food or tartary buckwheat leaves (18, 19). Fermentation with *A. niger* was found to increase the total phenolic content (TPC) and antioxidant properties of oats (20).

Currently, few reports are available on the result of solid-state fermentation (SSF) in terms of polyphenolic composition, antioxidant properties, and inhibition activities against metabolic syndrome-associated enzymes in AVL. In this study, *Monascus purpureus, A. oryzae*, and *A. niger* were used to investigate their influence on TPC and the total flavonoid content (TFC). Then, further research was done to optimize the conditions of fermentation of the best strain. In an ethanol extract and its three subfractions [EA, petroleum ether (PE), and water], the composition of individual phenolics, TPC and TFC, and the antioxidant and inhibitory activities against metabolic syndrome-associated enzymes in fermented or native AVL leaves were also discussed. Moreover, the correlation between TPC and TFC with antioxidant and inhibitory activities against metabolic syndrome-associated enzymes was investigated. This study provides a theoretical foundation for AVL fermented tea products with a stronger bioactivity by increasing the content of phenolics, especially quercetin and kaempferol.

## 2. Materials and methods

### 2.1. Microorganisms

In this research, *A. oryzae* (CICC40934, China Center of Industrial Culture Collection), *M. purpureus* (bio-67015, the inquiry network for microbial strains in China), and *A. niger* (CGMCC5.0809, China General Microbiological Culture Collection Center) were used to ferment AVL leaves. Before the experiment, these fungal strains were incubated on potato dextrose agar (PDA) plates at 28°C for 7 days for later use. To collect spores from these strains, the PDA surface was rinsed with 0.9% NaCl to obtain a spore suspension, which was then stored at 4°C for further experiments.

### 2.2. Substrate and SSF

*Apocynum venetum* L. leaves (30%, w/w, air-dried) and seed suspensions of *A. oryzae, M. purpureus*, and *A. niger* (8 log CFU/ml, 30%, v/w) with a water content of 40% (w/w) were used as substrate media for SSF. Before fermentation, the sterilized AVL leaf was obtained *via* autoclaving at 121°C for 20 min. Each group was divided into three parts. TPC and TFC of AVL were monitored at 3, 6, 9, and 12 days during fermentation.

### 2.3. Preparation of extracts

**Figure 1:**
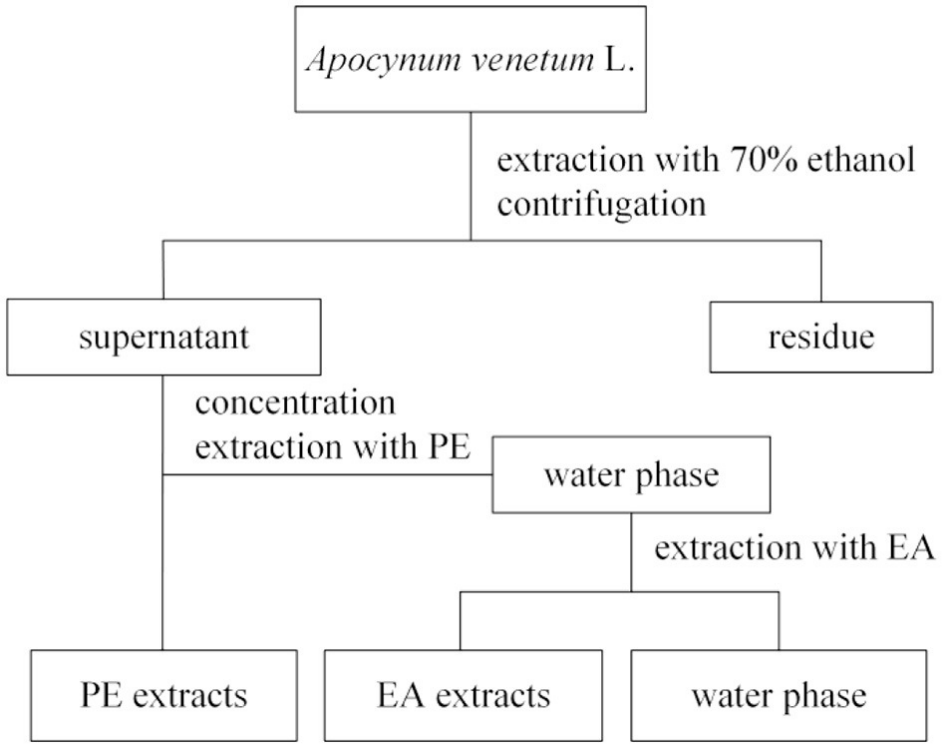
A procedure for ethanol extraction and separation of the petroleum ether (PE), ethyl acetate (EA), and water subfractions.

Approximately 1 g of dried AVL leaves (40 meshes) were extracted using 70% ethanol (1:20, v/v) in a water bath at 50°C for 1 h. The residue was extracted three times. Supernatants were evaporated in a rotary evaporator (45°C) until dry; then, the dry matter was dissolved again in 20 ml of the supernatant with 70% ethanol (v/v) to acquire the ethanol fractions. As was described in Figure 1, it was then dissolved in water and extracted with 30 ml of PE, and 30 ml of EA using a liquid–liquid partition of 1:1 (v/v). The ethanol, PE, EA, and water extracts were redissolved in dimethyl sulfoxide (DMSO) to yield the different phenolic fractions.

### 2.4. Determination of TPC and TFC

Samples (1 ml) were mixed with 1.5 ml of the Folin–Ciocalteu reagent at room temperature for ~3–8 min. Subsequently, 1 ml of Na_2_CO_3_ (20%, w/v) was added, and the total volume of 10 ml was filled with pure water. After incubation for 1 h, the optical density (OD) at 765 nm was measured. TPC was expressed as milligrams of gallic acid equivalents (GAE) per gram of dry weight (DW) or milliliters. These results were replicated at least three times with identical results.

The aluminum nitrate colormetric method was used to determine TFC, and rutin was used as standard. NaNO_2_ (1 ml, 5%, w/v) was added to samples (1 ml) and gently swirled for 5 min. Approximately 1 ml of Al(NO_3_)_3_ (10%, w/v) was added and maintained for 5 min, and 10 ml of NaOH (0.5 M) was added and allowed to react for 15 min. OD at 510 nm was measured. Experiments were performed three times. The TFC value was expressed as DW in milligrams of rutin equivalents (RTE) per gram or sample volume in milliliter.

### 2.5. Enzymatic assay

For the enzymatic assay, AVL fermented with *A. niger* was collected at different times (3, 6, 9, and 12 days). Wet weight substrate (3 g) was mixed with citric acid buffer (60 ml, pH 4.8) for the extraction of extracellular enzymes. The mixture was stirred in an ice bath at 120 revolutions per minute (rpm) for 6 h. The activities of carboxymethyl cellulase (CMCase), xylanase, and β-glucosidase were assayed, as mentioned in previous works (21, 22).

### 2.6. High-performance liquid chromatography analysis of EA subfractions

The samples were purified through a 0.22-μm syringe filter (Biosharp, China) for further high-performance liquid chromatography (HPLC). The components and concentration of PC in AVL were determined on an UltiMate™ 3000 RSLCnano System consisting of a Supersil AQ-C18 column (250 × 4.6 mm, 5 μm, Waters, China) as well as an RS variable wavelength detector (UltiMate™ 3000, DIONEX, USA). Acetonitrile solution and 0.2% phosphoric acid aqueous solution (v/v) were used as mobile phases A and B at a flow rate of 0.8 ml/min. The elution gradient concentration was set with 0–8 min, 5–12% solution A; 8–20 min, 12–18% solution A; 20–45 min, 18–22% solution A; 45–50 min, 22–35% solution A; and 50–60 min, 35–50% solution A. The other parameters of HPLC were operated: wavelength scanning detection (256 nm), a column temperature (35°C), and an injection volume (10 μl). The content of each standard in every group was calculated according to the standard curve (DW in mg/g). A standard curve was established using 100 μg/ml of chlorogenic acid, rutin, quercetin-3-*O*-galactoside, isoquercitrin, and kaempferol-3-*O*-glucose and 50 μg/ml of quercetin and kaempferol in methanol.

### 2.7. Assay of antioxidant activities and ferric reducing power

#### 2.7.1. ABTS·^+^ scavenging activity

The stable ABTS·^+^ (1:1, v/v) was generated by mixing the solution of 2.6 mM K_2_S_2_O_8_ and 7.4 mM ABTS·^+^, which was then reacted with antioxidants in samples. ABTS·^+^ and K_2_S_2_O_8_ mixed solution was reacted in a dark place at 12 h and diluted with ethanol (95%, v/v). The working solution was diluted with ethanol (95%, v/v) until the absorbance reached ~0.7. The final reaction mixture was combined with samples (200 μl) and 800 μl of ABTS·^+^ working solution. The mixture was incubated at 25°C for 6 min. The reaction was detected at 734 nm. Ascorbic acid was used as a positive control. The scavenging capacity was calculated:


$$\begin{array}{l} {\text{ABTS}}^{+}\text{ radical scavenging activity}=\frac{{\text{A}}_{0}-{\text{A}}_{1}}{{\text{A}}_{0}}\times \;100\%, \end{array}$$

where *A*_1_ and *A*_0_ are the ODs of the test sample and blank, respectively.

#### 2.7.2. DPPH· radical scavenging activity

Approximately 1 ml of samples and 1 ml of DPPH (0.2 mM) were mixed and maintained at 25°C for 10 min under darkness. Absorbance at 517 nm was measured. Ascorbic acid served as the positive control. The scavenging activity was calculated as follows:


$$\begin{array}{l} \text{DPPH radical scavenging activity}=1-\frac{{\text{A}}_{1}-{\text{A}}_{2}}{{\text{A}}_{0}}\times \;100\%, \end{array}$$

where *A*_0_, *A*_1_, and *A*_2_ are the OD values of the blank control, the test sample, and the sample control, respectively.

#### 2.7.3. Hydroxyl free radical (·OH) scavenging activity

Samples were mixed with FeSO_4_ (6 mM) and H_2_O_2_ (6 mM) solution (2 ml, 1:1:1, v/v/v) at room temperature for 10 min. Then, 2 ml of salicylic acid was added, and the absorbance at 510 nm was measured. Ascorbic acid served as a positive control. Percentage (%) inhibition of OH was used to express the results according to the following equation:


$$\begin{array}{l} \text{Hydroxyl radical scavenging activity}=\frac{{\text{A}}_{0}-{\text{A}}_{1}}{{\text{A}}_{0}}\times \;100\%, \end{array}$$

where *A*_1_ and *A*_0_ are the ODs of the reacted mixture with the test sample and blank control.

#### 2.7.4. Ferric reducing power

Approximately 1 ml of samples, 2.5 ml of polybutylene succinate (PBS) (0.2 M, pH 6.6), and 2.5 ml of K_3_[Fe(CN)_6_] (1%, m/v) were mixed in a sequence. After incubation for 20 min at 50°C, 1 ml of C_2_HCl_3_O_2_ (10%, m/v) was added and mixed. Then, 2.5 ml of the mentioned reaction mixture, 2.5 ml of pure water, and 0.5 ml of FeCl_3_ (1%, m/v) were mixed and kept for 30 min. Absorbance read at 700 nm was expressed as the ferric reducing power.

### 2.8. Inhibitory activity against metabolic syndrome-associated enzymes

#### 2.8.1. α-glucosidase inhibitory assay

Polybutylene succinate (25 mM, pH 6.9) was used to dissolve samples to 100 μg/ml. Approximately 0.2 ml of samples and 1 ml of α-glucosidase (1 × 10^−3^ mg/ml) were mixed and incubated at 37°C for 10 min. Then, 0.5 ml of p-nitrophenyl-α-D-glucopyranoside (p-NPG, 5 mM) was used to initiate the reaction for 10 min. Approximately 1 ml of Na_2_CO_3_ (0.1 M) was mixed to stop the reaction. The absorbance was measured at 405 nm. Samples were assayed three times. Acarbose served as the positive control. The inhibition ratio of α-glucosidase was calculated as follows:


$$\begin{array}{l} \text{The inhibition ratio}=1-\frac{{\text{A}}_{4}-{\text{A}}_{3}}{{\text{A}}_{2}-{\text{A}}_{1}}\times \;100\%, \end{array}$$

where *A*_4_, *A*_3_, *A*_2_, and *A*_1_ are the ODs of the tested group, the blank tested group, the control group, and the blank control group, respectively.

#### 2.8.2. Porcine pancreatic lipase inhibitory assay

Pure water was used to distill porcine pancreatic lipase type II to 5 mg/ml enzyme suspension. The supernatant was centrifuged at 6,000 × g at 4°C for 10 min, and it was recovered for further use. Pancreatic lipase solution, PBS (pH 7.4), and diluted samples (50 μl, 1:1:1, v/v/v) were blended and incubated at 37°C for 10 min. Then, 50 μl of *p*-nitrophenyl palmitate (*p*-NPB, 11.2 mM) was added at 37°C for 20 min, and the absorbance at 405 nm was measured. Samples were assayed in three duplicates. Orlistat served as the reference compound. The calculation formula is as follows:


$$\begin{array}{l} \text{The inhibition ratio}=1-\frac{{\text{A}}_{4}-{\text{A}}_{3}}{{\text{A}}_{2}-{\text{A}}_{1}}\times 100\% \end{array}$$

where *A*_4_, *A*_3_, *A*_2_, and *A*_1_ represent the ODs of the tested group, blank tested group, control group, and blank control group, respectively.

### 2.9. Statistical analysis

In this study, all data were reported as the mean ± standard deviation (SD) of three replications. One-way analysis of variance (ANOVA) was used to determine the difference analysis between values, and a *p*-value of < 0.05 was considered statistically significant. Correlation analysis was performed using a two-tailed Pearson's correlation test of GraphPad Prism version 8.0 software package.

## 3. Results

### 3.1. TPC and TFC of AVL fermented with *A. oryzae, M. purpureus*, and *A. niger*

The key step for the fermentation of AVL is to select suitable microorganisms. The effect of different fungi on fermentation was investigated by measuring TPC and TFC. In the present study, AVL was fermented with *A. oryzae, M. purpureus*, and *A. niger* for 6 days. Table 1 shows the observation of the highest TPC (1.67 mg GAE/ml) and TFC (0.44 mg RTE/ml) during fermentation with *A. niger*. Thus, *A. niger* was selected as the optimal fermentation fungus for subsequent experiments.

**Table 1:** Changes in the total phenolic content (TPC) and total flavonoid content (TFC) after fermentation of different strains.

	**TPC (mg GAE/ml)**	**TFC (mg RTE/ml)**
CK	1.26 ± 0.05^bc^	0.38 ± 0.01^b^
*A. oryzae*	1.12 ± 0.05^c^	0.29 ± 0.01^c^
*A. niger*	1.67 ± 0.01^a^	0.44 ± 0.01^a^
*M. purpureus*	1.31 ± 0.02 ^b^	0.38 ± 0.01^b^

Different superscript letters in the same column represent a significant difference (p < 0.05).

### 3.2. Determination of the optimal fermentation time of AVL with *A. niger*

To evaluate the optimal fermentation time, AVL was fermented with *A. niger* in four different stages (3, 6, 9, and 12 days). Table 2 shows a gradual increase in TPC and TFC with the extension of time before 9 days. On the 6th day, TPC and TFC of the *A. niger* treatment were 1.47 mg GAE/ml and 0.46 mg RTE/ml, respectively. Over 6 days, TPC and TFC were slightly decreased. Thus, the 6th day was the optimal fermentation period.

**Table 2:** Changes in TPC and TFC of *Apocynum venetum* L. (AVL) fermented with *Aspergillus niger* for 0, 3, 6, 9, and 12 days.

**Days**	**TPC (mg GAE/ml)**	**TFC (mg RTE/ml)**
0	1.14 ± 0.02^d^	0.40 ± 0.01^b^
3	1.42 ± 0.06^b^	0.46 ± 0.03^a^
6	1.47 ± 0.02^a^	0.46 ± 0.01^a^
9	1.44 ± 0.06^ab^	0.42 ± 0.01^b^
12	1.39 ± 0.03^c^	0.38 ± 0.00^c^

Different superscript letters in the same column represent a significant difference (p < 0.05).

### 3.3. CMCase, xylanase, and β-glucosidase activities of *A. niger* during the fermentation of AVL

To elucidate the relationship between enzyme activities produced by *A. niger* and bioactivity release of AVL during fermentation, we tracked the enzymatic activities of CMCase, xylanase, and β-glucosidase produced by *A. niger* during the fermentation process. Table 3 shows that CMCase (0.05 U/ml), xylanase (0.07 U/ml), and β-glucosidase (0.05 U/ml) activities were relatively low on the 3rd day of fermentation. On the 6th day of fermentation, the activities of CMCase and xylanase were significantly increased, and the activity of xylanase (0.21 U/ml) was relatively higher than CMCase (0.18 U/ml). On the 9th day of fermentation, the activity of xylanase (0.19 U/ml) had a slight decline, and the activity of CMCase (0.08 U/ml) was remarkably decreased. The β-glucosidase activity of *A. niger* was lower during the entire incubation period (0.05 U/ml at 3 days, 0.06 U/ml at 6 days, 0.08 U/ml at 9 days, and 0.05 U/ml at 12 days) as compared to CMCase and xylanase.

**Table 3:** Carboxymethyl cellulase (CMCase), xylanase, and β-glucosidase activities of *A. niger* during the AVL fermentation process at 3, 6, 9, and 12 days.

**Days**	**CMCase (U/ml)**	**Xylanase (U/ml)**	**β-glucosidase (U/ml)**
3	0.05 ± 0.00^b^	0.07 ± 0.00^b^	0.05 ± 0.00^b^
6	0.18 ± 0.06^a^	0.21 ± 0.02^a^	0.06 ± 0.01^b^
9	0.08 ± 0.01^b^	0.19 ± 0.01^a^	0.08 ± 0.04^a^
12	0.06 ± 0.01^b^	0.09 ± 0.01^b^	0.05 ± 0.00^b^

Different superscript letters in the same column represent a significant difference (p < 0.05).

### 3.4. TPC and TFC of the ethanol extract, PE, EA, and water subfraction of native (unfermented) and fermented AVL

**Figure 2:**
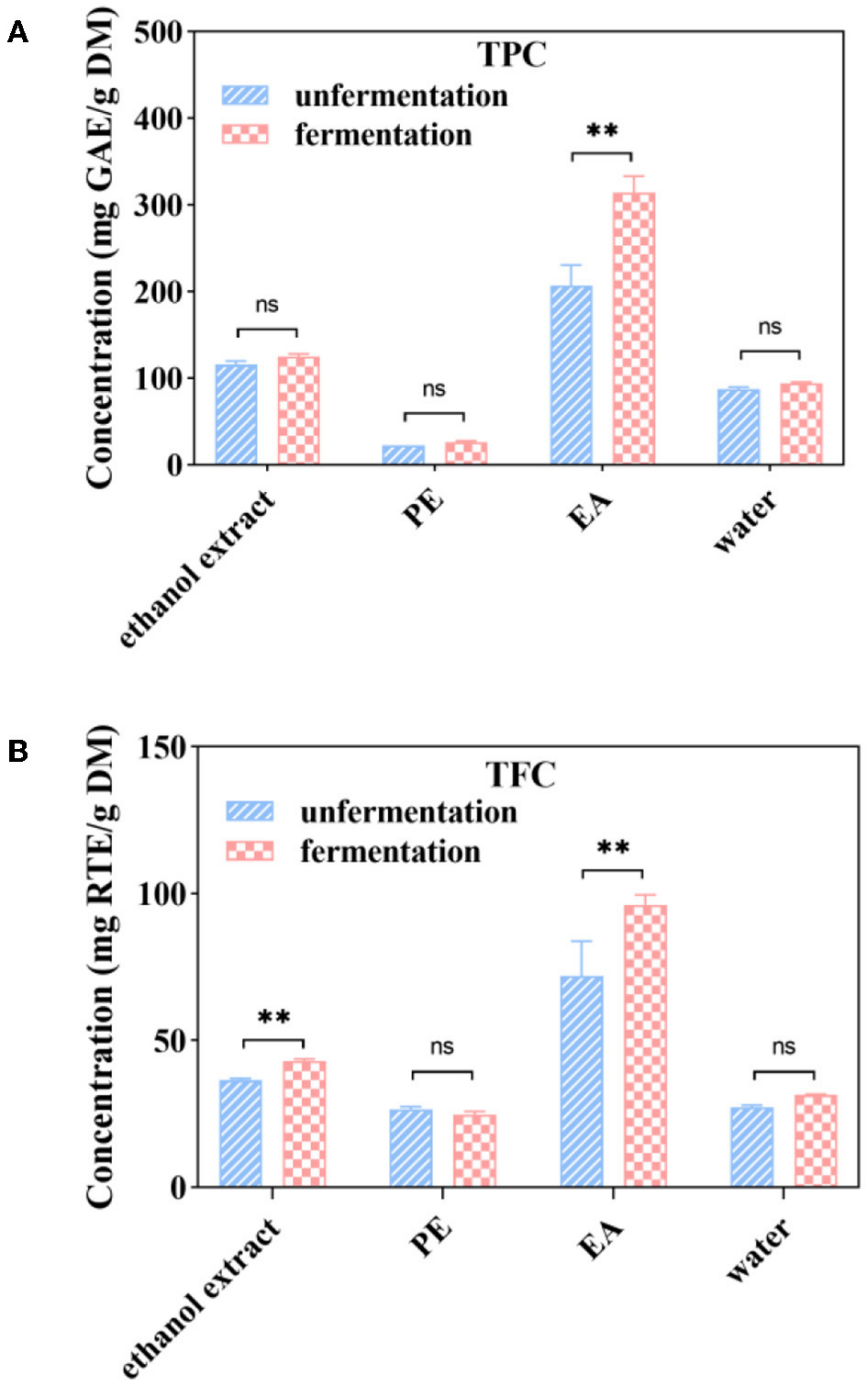
Soluble phenolic contents **(A)**, soluble flavonoid contents **(B)** and soluble phenolic extracts at different subfractions from *Apocynum venetum* L. (AVL) fermented with *A. niger*. ** *P* < 0.05.

The total phenolic content of ethanol extract, PE, EA, and water subfractions from native AVL is shown in Figure 2A. The EA subfraction showed higher TPC (206.84 mg GAE/g DM) than the water subfraction (87.41 mg GAE/g DM), and the PE subfraction had the lowest value (22.71 mg GAE/g DM). It was shown that fermentation significantly increased TPC in ethanol extract, EA, and water subfractions of AVL (Figure 2A). The EA subfraction of AVL fermented with *A. niger* (314.58 mg GAE/g DM) showed a more significant increase in TPC than that of native AVL (206.84 mg GAE/g DM). Only TPC of PE subfractions did not show any statistically significant differences between fermented and native AVL.

As shown in Figure 2B, the EA subfraction of native AVL showed the highest TFC of 71.93 mg RTE/g DM, followed by the water subfraction of 27.13 mg RTE/g DM, while TFC was not detected in PE subfractions (Figure 2). Compared with native AVL, TFC in the EA subfractions fermented with *A. niger* showed a remarkable increase of 33.49%. TFC in the water subfraction of native and fermented AVL showed no significant differences. In addition, TFC was not detected in PE subfractions of fermented AVL.

### 3.5. Comparison of antioxidant activities of ethanol extract, PE, EA, and water subfractions of native and fermented AVL

In this study, DPPH·, ABTS·^+^, hydroxyl radical scavenging activity, and ferric reducing power were used to measure the total antioxidant activities of ethanol extract, PE, EA, and water subfractions from native and fermented AVL.

#### 3.5.1. ABTS·^+^ radical scavenging activity

**Figure 3:**
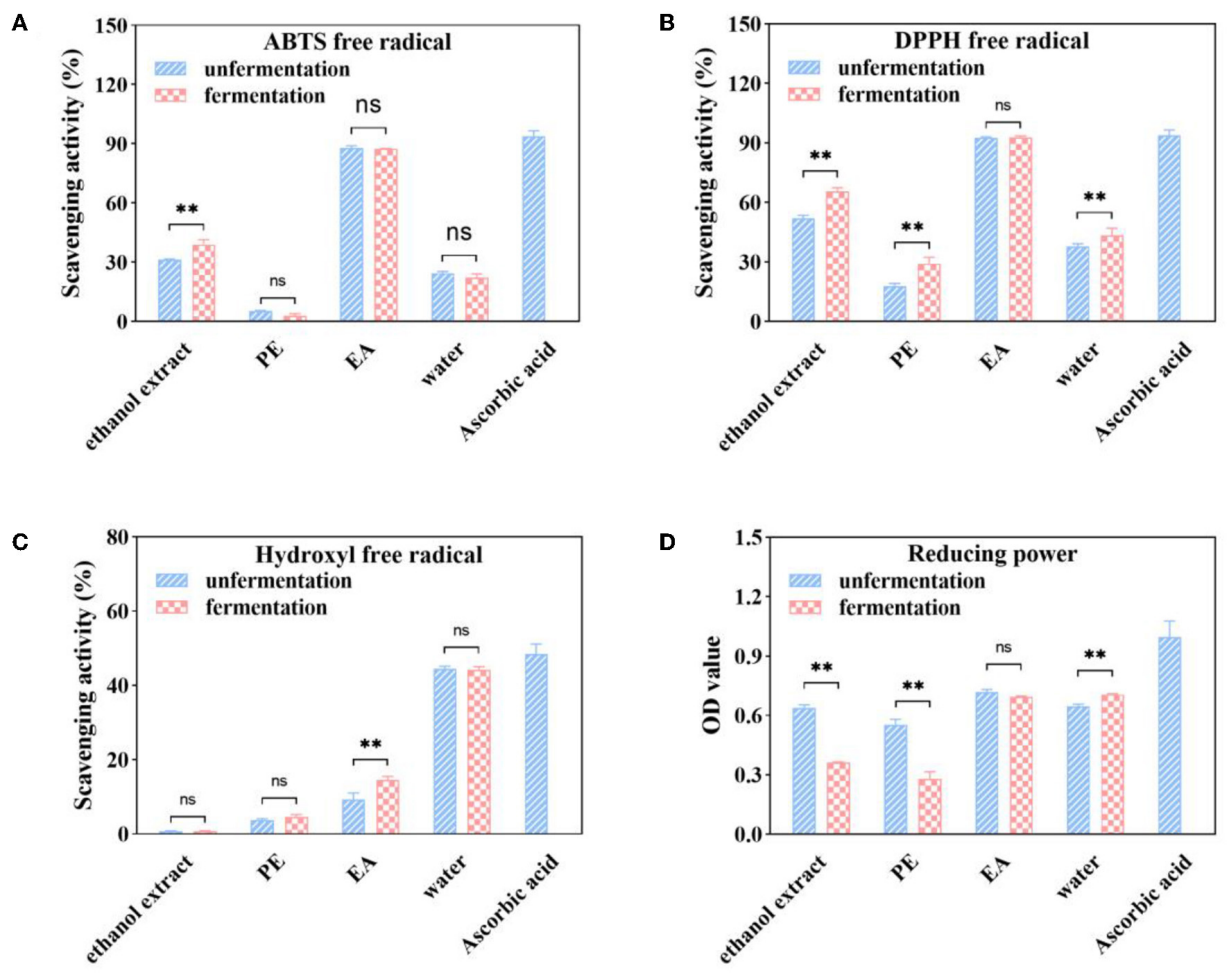
ABTS·^+^ **(A)**, DPPH **(B)**, hydroxyl **(C)**, and free radical scavenging activities and reducing power **(D)** of soluble phenolic extracts at different subfractions from AVL fermented with *A. niger* (100 μg/ml of ascorbic acid as a positive control). ** *P* < 0.05.

The scavenging ratio of the different subfractions from native and fermented AVL is shown in Figure 3A. Among the subfractions of unfermented AVL, the EA subfraction showed the highest ABTS·^+^ radical scavenging activity (87.81% with 100 μg/ml), followed by the water subfraction (24.44% with 100 μg/ml). The PE subfraction had the lowest ABTS·^+^ radical scavenging activity (5.28% with 100 μg/ml). Compared with a native AVL extract, the ABTS·^+^ radical scavenging ratio of the ethanol extract remarkably increased by 24.53% after *A. niger* fermentation, but the scavenging activity of EA subfractions showed no significant difference compared with the values of their native AVL.

#### 3.5.2. DPPH· radical scavenging activity

As shown in Figure 3B, the scavenging activity of DPPH· radical in untreated AVL was as follows: EA (92.67%) > water (37.88%) > PE (17.89%), which was consistent with TPC and ABTS·^+^ radical scavenging activity. Fermentation with *A. niger* was also found to have increased the DPPH·radical scavenging ratio in all of these four subfractions. The DPPH·radical scavenging ratio of the PE subfraction was improved by 63.17% over that of the untreated group. However, EA subfractions fermented with *A. niger* showed no differences with native AVL.

#### 3.5.3. Hydroxyl free radical (·OH) scavenging activity

As shown in Figure 3C, the water subfraction in untreated AVL had the highest (OH) scavenging activity (44.53% at a sample concentration of 1 mg/ml), followed by the (OH) scavenging activity of the EA subfraction (9.28%). The PE subfraction had the lowest value (3.76%). However, the TPC of water subfraction was lower than that of the EA subfraction (Figure 2A). It is indicated that·OH scavenging activity may not be related to the content of polyphenol. However, compared with native AVL, the·OH scavenging activity in EA subfractions was also increased by 56.27% after fermentation (Figure 3C).

#### 3.5.4. Ferric reducing power

Figure 3D shows that the EA subfraction has the best ferric-reducing activity (OD_700_ value = 0.6501 ± 0.012) followed by water subfraction (OD_700_ value = 0.6031 ± 0.011). In addition, we noted that ferric-reducing power is very low for the petroleum ether extract, which is the least polar extract (OD_700_ value = 0.5412 ± 0.018). The ethanol extract and PE subfraction of AVL have a reducing activity of ferric reducing power with a lower activity for fermentation with *A. niger* in comparison with a native group. However, the EA and water subfractions have no statistically significant differences compared to the fermented group in ferric-reducing power activity (*p* < 0.05).

### 3.6. Inhibitory activities of metabolic syndrome-associated enzymes

**Figure 4:**
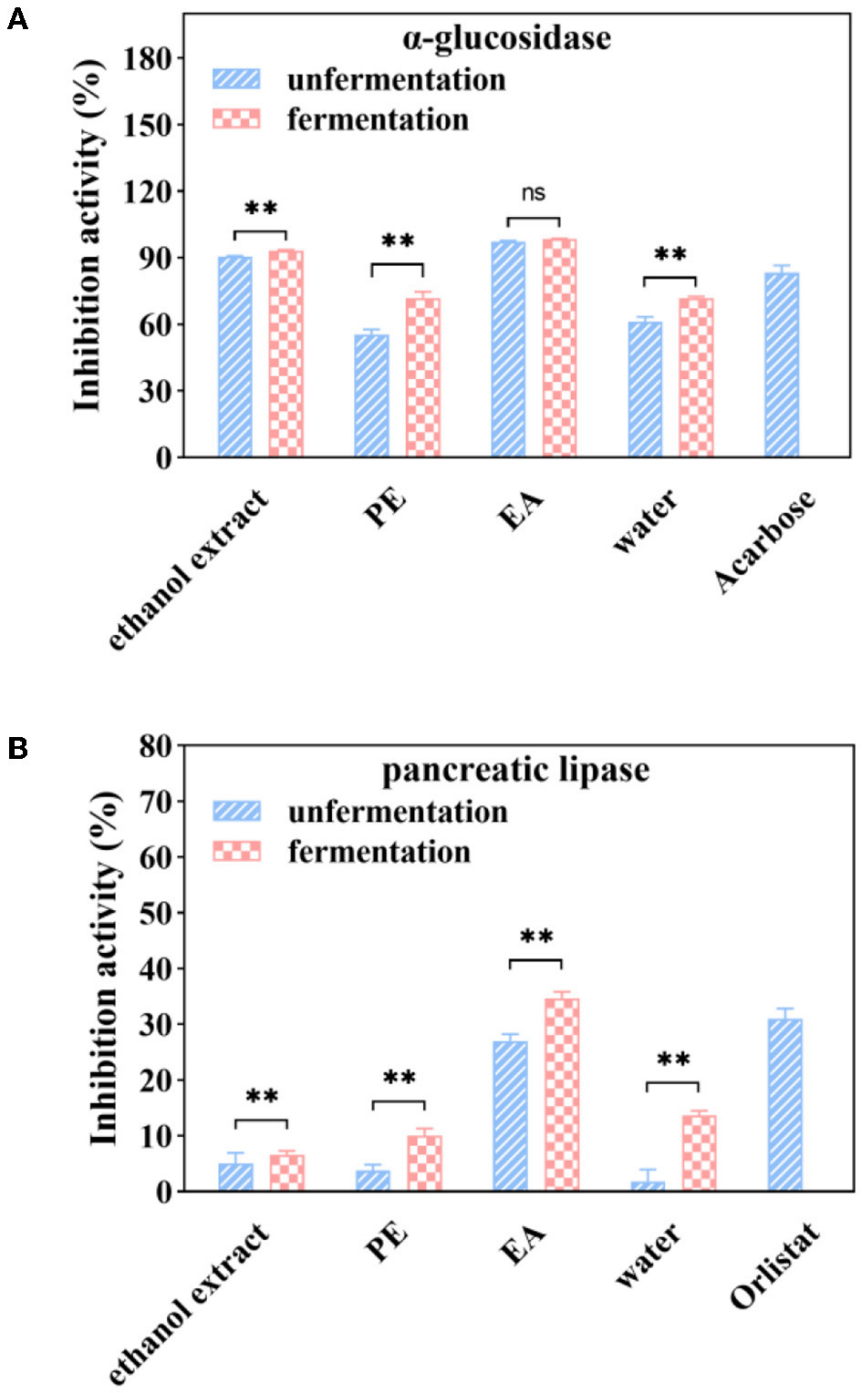
α-glucosidase **(A)** and pancreatic lipase **(B)** inhibition activities of soluble phenolic extracts at different subfractions from AVL fermented with *A. niger* (100 μg/ml of acarbose and 1 mg/ml of orlistat as positive controls). ** *P* < 0.05.

As shown in Figure 4A, inhibitory activities of α-glucosidase enzyme were 55.45, 97.48, and 61.27% for the PE, EA, and water subfractions at a sample concentration of 1 mg/ml, respectively. Inhibitory activities of PE and water subfractions were significantly increased by 29.64 and 16.00% after fermentation with *A. niger*. However, the inhibition of α-glucosidase was not significantly changed after fermentation in the EA subfraction.

Further experiment was performed to study the effect of fermentation on pancreatic lipase inhibition efficacy. It was shown that PE, EA, and water subfractions of pancreatic lipase inhibition activities were 4, 26, and 2%, respectively, at a sample concentration of 1 mg/ml (Figure 4B). Fermentation with *A. niger* enhanced the inhibitory efficiency of all subfractions on the inhibitory activities against porcine pancreatic lipase. After 6 days of fermentation, a significant increase (28%) in the inhibitory activities against pancreatic lipase was observed in the EA subfraction.

### 3.7. Changes in soluble phenolics in the EA subfraction of AVL fermented with *A. niger*

**Figure 5:**
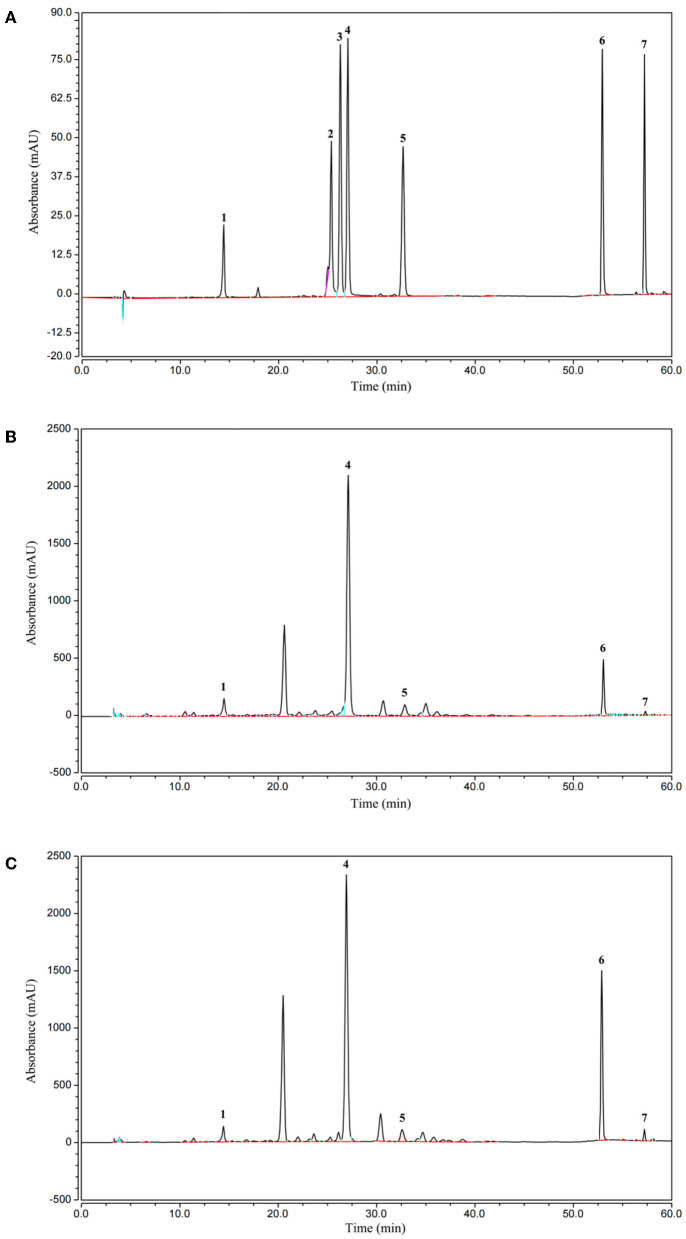
High-performance liquid chromatography (HPLC) chromatogram of the standard compounds **(A)**, native **(B)**, and fermented phenolics **(C)** in the EA subfraction of AVL, Peaks: 1, Chlorogenic acid; 2, Rutin; 3, quercetin-3-*O*-galactoside; 4, Isoquercitrin; 5, kaempferol-3-*O*-glucose; 6, Quercetin; 7, Kaempferol.

The effect of AVL fermented with *A. niger* on soluble phenolics was investigated. HPLC analyses were applied to the EA fraction, as this fraction had a higher TPC, TFC, DPPH, and ABTS·+ radical scavenging activity and ferric reducing power when compared to the ethanol, PE, and water subfractions. Quantities of seven compounds, including chlorogenic acid, rutin, quercetin-3-*O*-galactoside, isoquercitrin, kaempferol, quercetin, and kaempferol-3-*O*-glucose, were measured (Table 4; Figure 5). According to HPLC analyses, the composition of soluble phenolics was similar after *A. niger* fermentation, but their contents were significantly different. The soluble contents of most of the phenolics from AVL extracts were slightly increased due to fermentation. As shown in Figures 5A–C and Table 4, increases in phenolic components after fermentation were as follows: chlorogenic acid, 18%; rutin, 28%; quercetin-3-*O*-galactoside, 101%; isoquercitrin, 17%; kaempferol-3-*O*-glucose, 10%; quercetin 264%; and kaempferol 122%.

**Table 4:** Changes in individuals of soluble phenolics in the EA subfraction of AVL fermented with *A. niger*.

**Standard**	**Stage**	**Soluble phenolics (mg/g DM)**
chlorogenic acid	Native	31.27 ± 1.32
	Fermentation	36.89 ± 1.63
Rutin	Native	3.96 ± 0.18
	Fermentation	5.10 ± 0.21
quercetin-3-*O*-galactoside	Native	3.35 ± 0.13
	Fermentation	6.73 ± 0.32
Isoquercitrin	Native	162.74 ± 5.89
	Fermentation	190.38 ± 7.22
kaempferol-3-*O*-glucose	Native	14.02 ± 0.82
	Fermentation	15.46 ± 0.93
Quercetin	Native	14.58 ± 0.76
	Fermentation	53.14 ± 2.13
Kaempferol	Native	1.65 ± 0.11
	Fermentation	3.67 ± 0.19

### 3.8. Correlation analysis

A Pearson's test was carried out to evaluate correlations between TPC and TFC as well as bioactivities in the AVL extract. As shown in the correlation analysis results (Table 5), DPPH· and ABTS·^+^ scavenging activities were significantly positively correlated with TPC (*R*^2^ = 0.8926, *p* < 0.05 and *R*^2^ = 0.8612, *p* < 0.05) and TFC (*R*^2^ = 0.8175, *p* < 0.05 and *R*^2^ = 0.8778, *p* < 0.05). However, Table 4 displays no correlation between OH scavenging activity and TPC (*R*^2^ = 0.0003, *p* = 0.92) or TFC (*R*^2^ = 0.0245, *p* = 0.39). Positive correlations were observed between α-glucosidase inhibitory activity and TPC (*R*^2^ = 0.6181, *p* < 0.05) or TFC (*R*^2^ = 0.5750, *p* < 0.05). The pancreatic lipase inhibitory activity showed a stronger positive relationship with TFC (*R*^2^ = 0.8249, *p* < 0.05) than with TPC (*R*^2^ = 0.7334, *p* < 0.05).

**Table 5:** A correlation between biochemical composition and bioactivities.

	**Correlation coefficient**	
	**TPC**	**TFC**
DPPH·scavenging capacity	0.8926	0.8175
ABTS·^+^ scavenging capacity	0.8612	0.8778
OH scavenging activity	0.0003	0.0245
Ferric reducing power	0.2679	0.1890
α-glucosidase enzyme inhibitory activity	0.6181	0.5750
Pancreatic lipase inhibitory activity	0.7334	0.8249

Correlations between antioxidant activities and inhibitory activities against metabolic syndrome-associated enzymes in an AVL extract are presented in Table 6. The results indicated a positive correlation between antioxidant activities (DPPH· and ABTS·^+^ scavenging activity) and α-glucosidase (*R*^2^ = 0.8093, *p* < 0.05 and *R*^2^ = 0.6503, *p* < 0.05) and pancreatic lipase inhibitory activities (*R*^2^ = 0.6621, *p* < 0.05 and *R*^2^ = 0.7214, *p* < 0.05). The results also demonstrated no significantly positive correlation between antioxidant activities (OH scavenging activity and ferric reducing power) and inhibitory activities against metabolic syndrome-associated enzymes.

**Table 6:** A correlation between biochemical composition and antioxidant activities.

	**Correlative coefficient**	
	α**-glucosidase enzyme inhibitory activity**	**Pancreatic lipase inhibitory activity**
DPPH· scavenging capacity	0.8093	0.6621
ABTS·+ scavenging capacity	0.6503	0.7214
OH scavenging activity	0.1596	0.0016
Ferric reducing power	0.0258	0.1724

## 4. Discussion

Fermentation with filamentous fungi has been widely reported for the release of diverse polyphenolic compounds. *A. oryzae, M. purpureus*, and *A. niger* were recognized as safe fungi and employed in the preparation of foods (17, 20, 23). For example, Zhang et al. (19) used *A. niger* CJ-1 to ferment TBL. In the EA subfraction of *A. oryzae* fermented oats, caffeic and ferulic acids were increased by 2.7- and 5.5-fold, respectively, and chlorogenic and *p*-Coumaric acids also increased about two times (20). Fermentation with *M. anka* GIM 3.592 and *S. cerevisiae* GIM 2.139 enhanced the release of chlorogenic acid, rutin, and quercetin in guava leaves (23).

Based on the limitation of the type of enzymes secreted by microorganisms and the complexity of plant cell structures, suitable microorganisms should be selected for the fermentation of AVL. In the present study, *A. oryzae, M. purpureus*, and *A. niger* were applied in the fermentation of AVL leaves. Fermentation with *A. niger* showed a higher level of TPC than that with *M. purpureus*, while TPC fermented with *A. oryzae* had a lower value than that fermented with *A. niger* and *M. purpureus*. The data showed that an increase in TPC was related to the types of fungi. However, the biotransformation and degradation mechanism of polyphenol components of AVL fermented with *A. niger* has not been sufficiently clarified.

In the present study, one polyphenol conversion pathway was found by analyzing the differential metabolites in AVL fermented with *A. niger*. *A. niger* can induce β-glucosidase to hydrolyze 3-glycosidic linkage in isoquercitrin and kaempferol-3-*O*-glucoside, liberating quercetin and kaempferol. Compared with untreated AVL, the contents of quercetin and kaempferol were increased 3.64 and 2.22 times, respectively. In addition, after fermentation with *A. niger*, the contents of chlorogenic acid, rutin, quercetin-3-*O*-galactoside, isoquercitrin, kaempferol-3-*O*-glucoside, quercetin, and kaempferol were significantly increased due to fermentation with *A. niger*, which might attribute to the conversion of conjugated PC into a free polyphenol by *A. niger*. This microorganism could produce cellulase and xylanase to release PC conjugated with xylan, cellulose, or lignin (20, 24–26). Previous studies showed that there was a positive correlation between cellulase or xylanase activities and TPC (27–29).

It was reported that the major bioactive components of AVL mainly consisted of phenolic acids (chlorogenic acid and caffeic acid) and flavonoids (rutin, hyperin, isoquercitrin, quercetin, kaempferol quercetin-3-*O*-β-D-xylopyranoside, quercetin-3-*O*-α-L-arabinoside, and kaempferol-3-*O*-glucoside) (5, 30, 31). Phenolics are important native antioxidants in AVL. The present study showed that fermentation with *A. niger* significantly increased the DPPH·, ABTS·^+^, and OH radical scavenging activities of AVL. Meanwhile, fermentation with *A. niger* can greatly convert flavonoids (isoquercitrin and kaempferol-3-*O*-glucoside) into quercitrin and kaempferol. Previous reports confirmed that aglycone exhibited much higher antioxidant capacity than its glycoside derivatives (32, 33). For example, aglycone corresponding to quercetin showed a higher antioxidant activity than rutin (32). Compared with those lacking neutralizing free radicals, quercetin contained more hydroxyl groups and has a comparatively greater antioxidant potential (33). Hence, fermentation with *A. niger* cannot only further enhance the release of soluble phenolics from AVL but also transform glycoside compounds into aglycones with stronger antioxidant capacity.

In addition to antioxidant activities, AVL has been reported to have antihypertensive effects, cholesterol-lowering activity, and antidiabetic activity (2). In the present study, fermentation with *A. niger* showed stronger inhibition activities against α-glucosidase and pancreatic lipase than native AVL. Phenolic compounds were shown to perform *in vitro* inhibition activities against metabolic syndrome-associated enzymes like lipases, α-amylase, and α-glucosidase (34). The specific structural features of polyphenols had an effect on their inhibitory activities against these enzymes (35). For example, flavonoids had more hydroxyl groups than phenolic acids, and they showed higher inhibitory activities compared with phenolic acids (36). As mentioned earlier, increased contents of soluble phenolics, quercetin, and kaempferol in AVL fermented with *A. niger* could enhance their inhibitory activities against metabolic syndrome-associated enzymes.

## 5. Conclusion

*A. niger, M. purpureus*, and *A. oryzae* were used in the fermentation of AVL. AVL fermented with *A. niger* not only releases more free phenolics but also converts glycoside derivatives into aglycone. Compared with native AVL, fermentation with *A. niger* significantly increases free phenolics (particularly, quercetin and kaempferol). Among phenolics, isoquercitrin showed the highest level. Additionally, ethanol extracts from AVL following fermentation exhibited a much higher antioxidant activity and inhibitory efficacy toward α-glucosidase and pancreatic lipase. The present study provided guidance for obtaining tea processing methods with higher antioxidant, antidiabetic, and antihyperlipidemic activities.

## Data availability statement

The raw data supporting the conclusions of this article will be made available by the authors, without undue reservation.

## Author contributions

CC and DL: data curation, writing—original draft, and writing—review and editing. CX: software, data curation, and critical content review. YZ, WG, and ZZ: visualization and investigation. NL and YW: software and validation. CL, LY, and ZH: conceptualization, methodology, and software. CX and YP: supervision. All authors contributed to the article and approved the submitted version.
